# Prediction of relapses in patients with small vessel vasculitides: a multicenter cohort study on histopathological risk patterns

**DOI:** 10.1007/s00296-025-06049-1

**Published:** 2025-12-02

**Authors:** Kristian Vogt, Stefan Krämer, Teresa Maria Schreibing, Martin Busch, Tobias Schmitt, Sebastian Mosberger, Fabian Schumbrink, Thomas Neumann, Raoul Bergner, Thomas Rauen

**Affiliations:** 1 Department of Nephrology and Clinical Immunology, RWTH Aachen University Hospital, Pauwelsstraße 30, 52074 Aachen, Germany; 2 Department of Internal Medicine III, University Hospital Jena, Friedrich-Schiller University, Jena, Germany; 3 Department of Internal Medicine A, Nephrology and Rheumatology, Municipal Hospital Ludwigshafen, Ludwigshafen, Germany; 4 Department of Rheumatology and Immunology, Cantonal Hospital St. Gallen, St. Gallen, Switzerland

**Keywords:** Vasculitis, Granulomatosis with polyangiitis, Microscopic polyangiitis, Kidney diseases, Risk factors, Recurrence

## Abstract

Management of ANCA-associated vasculitis (AAV) has significantly improved, yet up to 40% of patients experience relapses, leading to worse long-term outcomes and organ damage. This multicenter cohort study investigated the prognostic value of histopathological patterns in renal AAV for predicting relapse risk and treatment response. We retrospectively analyzed 264 patients with newly diagnosed granulomatosis with polyangiitis (GPA) or microscopic polyangiitis (MPA) with renal involvement recruited at four tertiary rheumatology and/or nephrology centers. Baseline clinical data, disease activity scores, and kidney biopsy findings were assessed. Patients were followed for at least twelve months. An overall relapse was defined as renewed disease activity requiring changes in maintenance therapy, initiation of a new induction regimen, or an increase in prednisone dosage > 10 mg/day. A severe relapse required new immunosuppressive induction therapy. After a median follow-up of twelve months the renal domain of the Birmingham Vasculitis Activity Score (BVAS) and its subcategories were evaluated. AAV patients with moderate-to-high (> 25%) interstitial fibrosis and tubular atrophy (IFTA) had a lower risk for both, overall and severe relapses. Severe relapses occurred more frequently in older and male patients. Furthermore, we identified glomerular sclerosis (≥ 50%) to be the strongest predictor for ongoing activity in the renal BVAS domain. Histopathological features do not only help to predict renal recovery and need for dialysis but also to forecast relapse risk and renal BVAS activity. Incorporating these patterns into clinical decision-making could enable more personalized therapy approaches, highlighting the need for prospective validation.

**Supplementary Information:**

The online version contains supplementary material available at 10.1007/s00296-025-06049-1.

## Introduction

Antineutrophil cytoplasmic antibody (ANCA)-associated vasculitides (AAV) comprise a spectrum of rare autoimmune diseases with estimated prevalence rates of 30–218/million [1].

According to the revised Chapel Hill Consensus Conference criteria from 2012, AAV predominantly affect small vessels comprising the entities of granulomatosis with polyangiitis (GPA), microscopic polyangiitis (MPA) and eosinophilic granulomatosis with polyangiitis (EGPA) [2]. Renal involvement as evidenced by the characteristic pattern of a pauci-immune extracapillary glomerulonephritis in kidney biopsy is common and affects about 73% of AAV patients [3]. It often marks the most threatening organ involvement that may lead to chronic kidney disease (CKD) and end-stage kidney disease (ESKD) in about 30% of patients [4, 5]. In 2010, Berden et al*.* proposed histopathological classification criteria for ANCA-associated glomerulonephritis classifying sclerotic, crescentic, mixed and focal subtypes [6]. Over time, different classification systems were established and several risk stratification modalities were developed [7]. The recently developed ANCA Kidney Risk Score (AKRiS) incorporated the degree of interstitial fibrosis and tubular atrophy (IFTA) and demonstrated promising results in predicting the 3-year risk of ESKD development in AAV patients [8]. The latest AAV classification criteria were published in 2022 by the American College of Rheumatology (ACR) and the European Alliance for Associations for Rheumatology (EULAR) [9, 10]. Proteinase 3 (PR3) and myeloperoxidase (MPO) specific ELISA findings play a crucial role for the classification of GPA or MPA in the updated criteria.

The therapeutic armamentarium broadened significantly over the last decades and includes well established substances like prednisone and methotrexate as well as cyclophosphamide (CYC) [11] and rituximab (RTX) [12] for severe, organ-threatening cases, or new drug classes like the complement C5 inhibitor avacopan [13]. Treatment is clustered into an immunosuppressive induction and a subsequent maintenance phase. Over the last decade, RTX has evolved as corner stone for both treatment phases and is first-line recommendation in multiple current guidelines for the management of AAV patients [14, 15]. Despite these advances in the immunosuppressive treatment regimen, relapsing disease courses are still common and affect up to 40% in GPA and 25% in MPA patients [3, 16–18]. Common risk factors in relapse prediction encompass increasing ANCA, particularly PR3 titers [17, 19–21] and lung involvement [16, 20]. Previously, we have reported that distinct pulmonary patterns, like ground glass opacities increase the risk for relapse [18]. Relapsing patients often display more organ damage [22] and renal relapses are associated with a higher risk to develop ESKD [23]. With the present analysis, we aimed to analyze risk factors for relapse and for ongoing disease activity, especially including histological patterns for risk stratification, derived from an multicentric European cohort of GPA and MPA patients.

## Methods

### Study design, patients and data acquisition

Baseline characteristics and inclusion criteria of the study population of GPA and MPA patients have been described before [3]. Briefly, we included newly diagnosed GPA and MPA cases from three German (Aachen, Jena, Ludwigshafen) and one Swiss (St. Gallen) tertiary rheumatology and/or nephrology referral centers between 1999 and 2022 with available follow-up data of at least 1 year upon diagnosis of AAV. Disease entities were re-classified based on the 2022 ACR/EULAR criteria [24]. Exclusion criteria were insufficient information about initial diagnosis, follow-up data under one year and patients without MPA or GPA. We retrospectively analyzed baseline parameters including organ involvement, Birmingham Vasculitis Activity Score (BVAS, version 3), laboratory and histological findings at the time of diagnosis, therapeutic agents for immunosuppressive induction treatment and outcome as well as organ damage measures. Data were collected from records of routine clinical visits also including hospitalizations as deposited in the medical information systems at each center.

Kidney biopsies obtained at the time of initial diagnosis were analyzed assessing the proportion of glomeruli with crescent formation, the degrees of necrosis, sclerosis, interstitial fibrosis and tubular atrophy (IFTA), and the percentage of unaltered glomeruli. Results are reported as percentages of the total number of glomeruli in each biopsy. Patients were stratified into three groups based on the extent of glomerular alterations: low alterations (≤ 25%), moderate-to-high alterations (> 25%), and high alterations (≥ 50%). A high degree of histopathological alteration aligns with the clusters defined by the 2010 Berden classification: focal class (≥ 50% normal glomeruli), crescentic class (≥ 50% glomeruli with cellular crescents), sclerotic class (≥ 50% globally sclerotic glomeruli), and mixed class (no single pattern exceeding 50%) [6].

An overall relapse was defined as renewed disease activity requiring change in immunosuppressive maintenance therapy, initiation of a new induction regimen, or an increase in prednisone dosage > 10 mg/day. A severe relapse event was defined as relapse that required new immunosuppressive induction therapy. This definition corresponds the “major” relapse category proposed by the EULAR recommendations for conducting clinical studies and/or clinical trials in systemic vasculitis [25]. A kidney-related relapse was defined by an enhanced activity recorded within the renal BVAS score documenting new hypertension, proteinuria > 1 g/day, new hematuria (active sediment) or rising serum creatine (due to active glomerulonephritis). Dialysis patients were excluded from the analysis of kidney-related relapses. Ongoing activity in the renal BVAS domain was defined as a score > 0 after a median of twelve months.

### Statistical analysis

Statistical analysis and graphical illustration were performed using R-Studio (version 4.2.3). For group comparison of dichotomic features x^2^- or Fisher’s exact test were used. Metric values were checked for normal distribution with Shapiro–Wilk test with subsequent Students` t-test or Mann–Whitney U-tests. For predictor analyzes, we performed logistic regression models. To assess multi-collinearity among the predictor variables in the multivariable model, variance inflation factor (VIF) values were calculated. The Box-Tidwell test was conducted to assess the assumption of linearity between continuous predictor variables and the log-odds of the outcome variable. If event time data were available, hazard ratios (HR) were calculated by using Cox proportional hazard models. Significant candidate parameters identified in preceding univariate Cox analyses, as well as clinically relevant covariates such as age, gender, GPA subtype, baseline eGFR, baseline BVAS score, and RTX as induction treatment, were included in the subsequent multivariate analysis. For Cox regression modeling, we applied a stepwise backward and forward selection based on the Akaike information criterion (AIC) to optimize model selection and minimize the risk of overfitting. Proportional hazard assumption was checked by using Schoenfeld residuals. In case of violation the variable was modeled as a time-dependent covariate or included as a stratification factor.

Glomerular filtration rate (GFR) was estimated using the 2009 Chronic Kidney Disease Epidemiology Collaboration (CKD-EPI) formula. Metric means are given ± standard deviation (SD). Median values are shown with interquartile ranges (IQR). P-values < 0.05 were regarded significant.

## Results

### Prediction of relapses

**Fig. 1:**
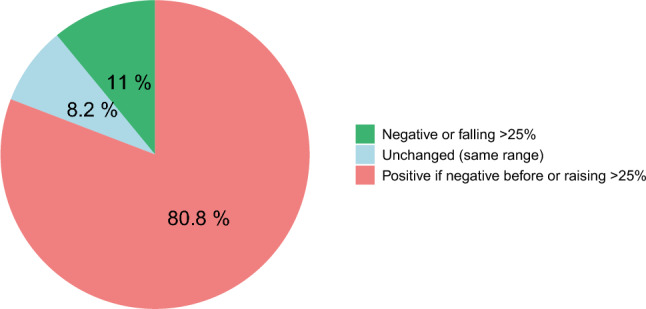
Change of ANCA titers during relapse. *ANCA* Anti-neutrophil cytoplasmic antibodies

In our overall AAV cohort (n = 358), renal involvement was more common in MPA patients (84.9%) as compared to those with GPA (68.3%) [3]. For the present analysis, we only included AAV patients with kidney involvement (n = 264) according to BVAS entries (Suppl. Fig. 1). Baseline characteristics, kidney biopsy information and outcomes of this cohort are provided in Table 1. Kidney biopsy results were available in 241 of these patients (i.e. 91.3%). Thirty-four (12.9%) of patients had pre-existing CKD, 29 (11%) had concomitant diabetes. Median follow-up observation in patients with renal involvement was 51.6 (IQR 28.5–96.3) months. Eighty-five patients (32.2%) exhibited at least one overall relapse, 45 (17.1%) had a severe relapse requiring a new course of immunosuppressive induction treatment. Nineteen patients (7.2%) developed multiple relapses. Forty-nine (22.3%) patients experienced kidney-related relapses. Median time to first overall relapse was 35.1 (IQR 13.9–59.3) months. At the time of an overall relapse, most patients (80.8%) had an increase in their specific ANCA autoantibody titers as assessed by ELISA readings with changes from negative to detectable values or increases by at least 25% as compared to prior assessments. Only 8.2% of patients experienced a relapse without a preceding increase in ANCA titers, while 15% either remained ANCA-negative or showed a decrease in titers by at least 25% (Fig. 1).

**Table 1:** Baseline characteristics, kidney biopsy information and outcomes of included AAV patients (all with kidney involvement according to baseline BVAS)

Characteristics	n = 264
Male gender, n (%)	145 (54.9)
Age (years), median (IQR)	62 (52–70)
BMI (kg/m^2^), median (IQR)	26.5 (23.8–29.7)
Follow-up time (months), median (IQR)	51.6 (28.5–96.3)
Baseline creatinine (µmol/L), median (IQR)	194.5 (100.3–346.7)
Baseline eGFR (mL/min), median (IQR)	26.6 (13.7–58.7)
Proteinuria (mg/day) at baseline (median, IQR)	1123.5 (589.3–2349.3)
CRP (mg/L) at baseline, median (IQR)	59 (11–150)
Baseline BVAS, median (IQR)	17 (12.0–20.3)
AAV subtype, n (%)	
GPA	143 (54.2)
MPA	118 (44.7)
Comorbidities, n (%)	
Diabetes	29 (11.0)
Pre-existing CKD	34 (12.9)
Organ involvement (BVAS), n (%)	
General	156 (59.1)
Cutaneous	33 (12.5)
Mucous membranes/eyes	35 (13.3)
ENT	78 (29.5)
Chest	129 (48.9)
Cardiovascular	11 (4.2)
Abdominal	9 (3.4)
Renal	264 (100.0)
Nervous system	38 (14.4)
Outcomes	
Overall relapses, n (%)	85 (32.2)
Kidney-related relapses, n (%)	49 (22.3)
Months to first relapse, median (IQR)	35.1 (13.9–59.3)
Multiple relapses (> 1), n (%)	19 (7.2)
Severe relapses, n (%)	45 (17.1)
Infections, n (%)	102 (38.6)
Deaths, n (%)	21 (8.0)
Need for dialysis, n (%)	44 (16.7)
Last recorded VDI, median (IQR)	2 (1–4)
Activity in renal BVAS domain after 12 months, n (%)	27 (10.3)
Hypertension	5 (18.5)
Proteinuria	19 (70.4)
Hematuria	8 (29.6)
> 30% rise in creatinine or > 25% fall in creatinine clearance	9 (33.3)
Kidney biopsy information (available in 241 cases, 91.3%)	

	All	Relapsing	Non-relapsing
Glomeruli count (mean, SD)	18.9 (12.4)	20.6 (12.4)	18.0 (10.1)
Glomeruli with necrosis (%, mean, SD)	20.2 (24.1)	22.5 (27.2)	19.0 (22.3)
Glomeruli with crescents (%, mean, SD)	15.7 (25.3)	21.1 (29.0)	12.7 (22.6)
Glomeruli with sclerosis (%, mean, SD)	25.8 (26.0)	21.6 (12.5)	28.0 (26.2)
IFTA (%, mean, SD)	23.2 (22.8)	17.5 (20.8)	25.9 (23.3)
Normal glomeruli (%, mean, SD)	43.3 (28.8)	42.8 (30.1)	43.6 (28.1)
Induction Treatment, n (%)			
CYC	197 (74.6)		
RTX	59 (22.4)		
Plasmapheresis	64 (24.2)		

*BMI*  body mass index, *eGFR*  estimated glomerular filtration rate, *CRP*  C-reactive protein, *BVAS*  Birmingham Vasculitis Activity Score, *GPA*  granulomatosis with polyangiitis, *MPA*  microscopic polyangiitis, *CKD*  chronic kidney disease, *ENT*  ear, nose and throat, *VDI*  Vasculitis Damage Index, *IFTA*  interstitial fibrosis and tubular atrophy, *CYC*  cyclophosphamide, *RTX*  rituximab

Patients experiencing at least one overall relapse were mainly men (p = 0.039), had more ENT (p = 0.033), abdominal involvement (p = 0.033) and GPA (vs. MPA, p = 0.049), whereas non-relapsing patients had significantly more often moderate-to-high IFTA > 25% (p = 0.046) in the biopsy, pre-existing CKD (p = 0.032) and an RTX-based induction regimen (p = 0.007) (Suppl. Table 1).

**Fig. 2:**
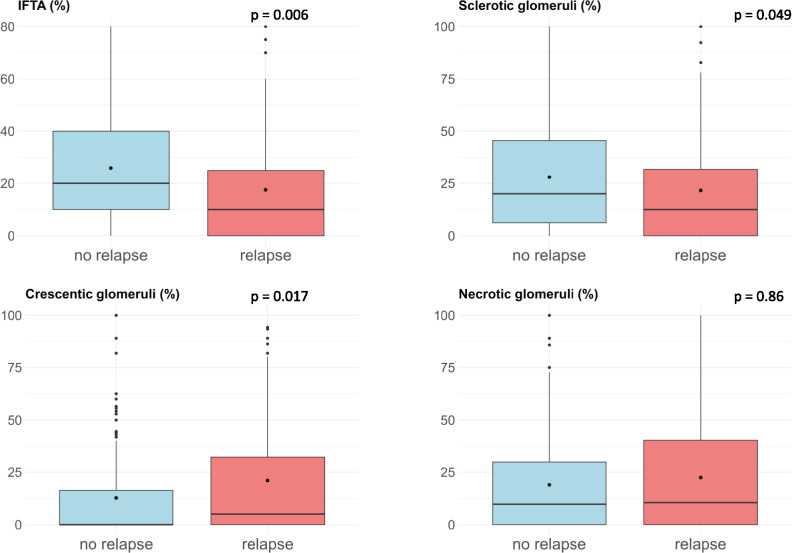
Histopathological patterns from kidney samples in relapse and non-relapse patients. Line indicating median, dot indicates mean. *IFTA* interstitial fibrosis and tubular atrophy

We then analyzed the distribution of individual histopathological patterns at baseline between relapsing and non-relapsing patients (Fig. 2). The rate of crescent formation was higher among relapsing patients in comparison with non-relapsing individuals (21.1% vs. 12.7%, p = 0.017), whereas the presence of sclerotic glomeruli (21.6% vs. 28.0%, p = 0.049) and the percentage of IFTA (17.5% vs. 25.9%, p = 0.006) were lower in patients with recorded relapse.

**Fig. 3:**
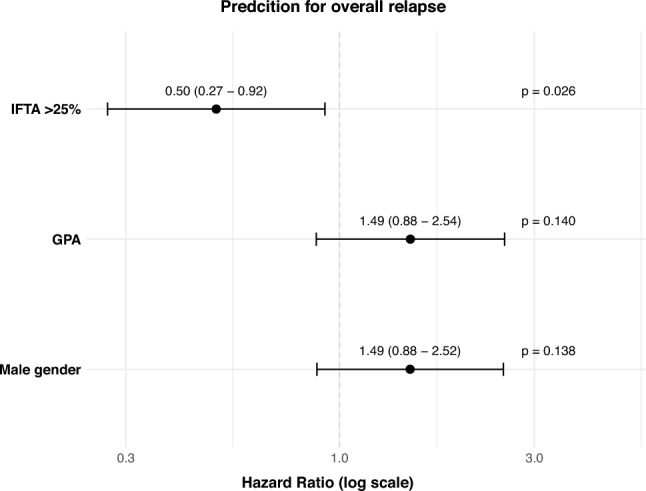
Forest plot displays hazard ratio for different predictors in a multivariate Cox regression model for the event overall relapse. Predictors included were significant variables from univariate analyses, plus key covariates: age, male gender, GPA subtype, baseline eGFR, baseline BVAS score, and RTX induction treatment. Model selection utilized stepwise backward/forward selection based on Akaike information criterion (AIC) to optimize predictive performance. Model’s discrimination was moderate, with a concordance index (C-index) of 0.59 (standard error = 0.037). Overall model fit was statistically significant as indicated by the likelihood ratio test (p = 0.01). *GPA* granulomatosis with polyangiitis, *eGFR* estimated glomerula filtration rate, *BVAS* Birmingham Vasculitis Activity Score, *RTX* rituximab, *IFTA* interstitial fibrosis and tubular atrophy

Using univariate Cox regression analysis, we identified moderate-to high- (HR 0.48), high IFTA (HR 0.43) and mucous membranes/eyes involvement (HR 1.82) as significant predictors for the occurrence of an overall relapse (Suppl. Table 2). For subsequent multivariate analysis we included these parameters in addition to key covariates including age, gender, GPA subtype, baseline eGFR, baseline BVAS score, and RTX induction treatment. In the final optimized model moderate-to high IFTA (HR 0.50) appeared to associate with a reduced risk for an overall relapse (Fig. 3).

### Prediction of kidney-related and severe relapses

Subsequently, we analyzed the impact of histopathological patterns on kidney-related relapses, defined as a relapse with reported activity in the renal domain of the BVAS score. For this analysis, we excluded patients requiring dialysis, since it is unlikely for these individuals to fulfill criteria to record renal BVAS activity. Patients with high-degree IFTA were less prone to develop kidney-related relapses, yet this did not meet the criteria of statistical significance (HR 0.69, 95%-CI 0.341–1.414, p = 0.315), alike patients with necrotic glomeruli (HR 0.47, 95%-CI 0.146–1.538, p = 0.214). Patients with ≥ 50% normal glomeruli (focal class) were at highest risk for kidney-related relapses (HR 1.30, 95%-CI 0.718–2.356, p = 0.387) however this association was also not significant (data not illustrated).

**Fig. 4:**
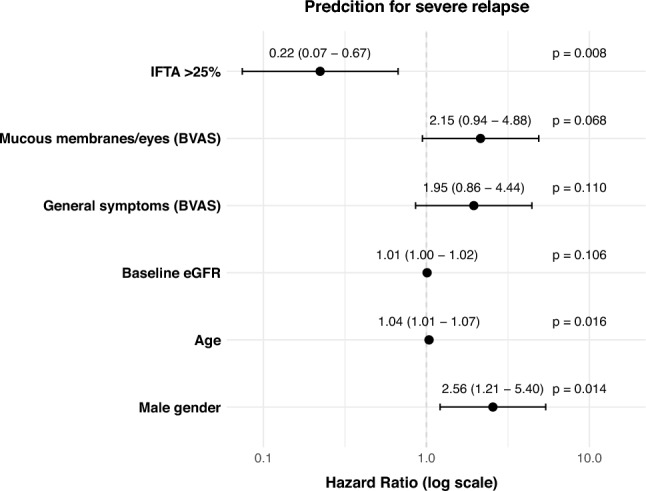
Forest plot illustrating the hazard ratios (HR) for different predictors in a multivariate Cox regression model for the event severe relapse. Predictors included were significant variables from univariate analyses, plus key covariates: age, male gender, GPA subtype, baseline eGFR, baseline BVAS score, and RTX induction treatment. Model selection utilized stepwise backward/forward selection based on Akaike information criterion (AIC) to optimize predictive performance. The model showed a concordance index (C-index) of 0.73 (SE = 0.048), indicating good discrimination ability. Overall model fit was statistically significant, as demonstrated by the likelihood ratio test (p < 0.001). *GPA* granulomatosis with polyangiitis, *eGFR* estimated glomerula filtration rate, *BVAS* Birmingham Vasculitis Activity Score, *RTX* rituximab, *IFTA* interstitial fibrosis and tubular atrophy

Univariate Cox regression analyzes identified moderate-to high (HR 0.23) and high IFTA (HR 0.11), activity in the general BVAS domain (HR 2.01), mucous membranes/eyes involvement (HR 0.010) and pre-existing hypertension (HR 0.39) as significant predictors for severe relapse events. Again, for multivariate analysis we included these parameters plus same covariates as before. The optimized Cox model showed significant results for moderate-to high IFTA (HR 0.22), age (HR 1.04) and male gender (HR 2.56) (Fig. 4).

### Persistent activity in the renal BVAS domain

After a median follow-up of twelve months, 27 patients (10.3%) still had signs of ongoing activity in the renal BVAS domain. Most commonly patients had proteinuria (70.3%) followed by > 30% rise in creatinine or > 25% fall in creatinine clearance (33.3%), hematuria (29.6%) and hypertension (18.5%) (Table 1). High glomerulosclerosis (OR 3.97), high crescent formation (OR 3.00) and ≥ 50% unaltered glomeruli (OR 0.31) significantly impacted persistent renal BVAS activity. Subsequent multivariate analysis, adjusted for relevant covariates as before, identified high-degree of glomerulosclerosis (OR 3.82) to be an independent predictor for an increased activity in the renal BVAS domain (Table 2).

**Table 2:** Multivariate logistic regression model for ongoing disease activity in the renal BVAS domain after a median of twelve months

Predictors	Odds Ratio	95%-CI	P value
Glom. sclerosis ≥ 50%	3.821	1.109–13.795	0.035*
Glom. crescents ≥ 50%	3.478	0.831–14.593	0.082
Normal glomeruli ≥ 50%	0.882	0.209–3.578	0.859
GPA	0.701	0.233–2.05	0.517
Induction with RTX	1.212	0.328–4.052	0.762
Age	0.973	0.933–1.015	0.200
Baseline eGFR	0.989	0.963–1.012	0.372
Male gender	0.954	0.321- 2.953	0.933
Baseline BVAS Score	0.966	0.865–1.069	0.516

Predictors included were significant variables from univariate analyses, plus key covariates (age, male gender, GPA subtype, baseline eGFR, baseline BVAS score, and RTX induction treatment)

*GPA* granulomatosis with polyangiitis, *eGFR* estimated glomerular filtration rate, *BVAS* Birmingham Vasculitis Activity Score, *RTX* rituximab

## Discussion

In this multicentric cohort of AAV patients with kidney involvement, we were able to link several demographic, histologic and treatment factors to an increased relapse risk. These insights significantly complement and extend previous findings from our AAV cohorts [3, 18]. Our results align with prior studies from other groups, indicating that GPA patients had numerically more overall relapses as compared to their MPA counterparts [16, 17, 20, 26], although we did not find a significant impact of the AAV subtype in different regression models. Yet, we found that an IFTA > 25% was associated with a reduced risk of both, overall and severe relapses. Additionally, older age and male gender were linked to a higher risk for severe relapse events. Finally, a high fraction of sclerotic glomeruli (≥ 50%) emerged as the strongest predictor for ongoing activity in the renal BVAS domain. Notably we did not observe significant factors predicting kidney-related relapses. Assessment of kidney-related relapses may be biased due to our definition of disease activity, which relies on increased renal BVAS scores reflecting new, worsened or persistent features within the past three months, potentially overlooking prior disease activity. The optimal definition of renal relapse in patients with ANCA-associated vasculitis (AAV) remains a matter of ongoing debate [27]. Current leading guidelines, such as those issued by KDIGO [15], employ a broad definition, characterizing relapse as the reappearance or worsening of disease activity after a period of partial or complete remission.

Most of our patients showed increasing ANCA autoantibody titers or a conversion from negative to positive at the time of relapse. Raising ANCA titers are a well described risk factor for relapse [21, 26, 28]. Nevertheless, approximately one in five patients exhibited a relapse despite unchanged or negative ANCA titer.

In AAV patients, there are only few studies that investigated the role of different histopathological patterns and the risk for relapse. One study reported that the proportion of sclerosed glomeruli and lack of interstitial infiltrates are predictive for relapse [29]. In this study, IFTA did not have a significant impact on kidney-related relapse events, but the degree of fibrosis weakly correlated with the extent of interstitial infiltrates. Thus, the authors hypothesized that fewer numbers of functional glomeruli made the kidney more prone for relapse events. By contrast, our data demonstrated moderate-to-high IFTA to be linked to a reduced HR of 0.50 for overall relapses and a HR of 0.22 for severe relapses, indicating that chronic damage patterns reduced relapse risk. This might be explained by the reduced capacity for inflammatory flares, which results from both the decreased amount of functional renal parenchyma and a disturbed immune cell response due to advanced tissue fibrosis. The interplay between inflammation and fibrosis is complex and putatively bidirectional. Tissue inflammation is a well-established predictor for the development and progression of kidney disease [30–32]. Moreover, inflammatory activity can be ongoing in fibrotic areas. In kidney transplant recipients for example, persistent inflammation within fibrotic areas is itself associated with poorer outcomes [33].

A possibly reduced relapse risk in patients with chronic damage patterns is additionally supported by a numerically reduced fraction of patients with pre-existing CKD in the relapsing group (5.9 vs 16.2%) and a reduced HR (0.39) for patients with pre-existing hypertension in the univariate Cox regression for predicting severe relapses. Especially hypertension is an established risk factor for developing CKD [34, 35]. In line with this, it has been reported that increased serum creatinine levels > 200 µmol/L at the time of diagnosis are strongly associated with a reduced risk of relapses [17, 36]. Alike, we observed a greater number of sclerotic glomeruli among non-relapsing patients (28.04 vs. 21.58%).

The presence of ≥ 50% sclerotic glomeruli was the strongest predictor for ongoing activity in the renal BVAS domain in our multivariate analysis, with proteinuria being the most commonly attributed BVAS item. The implication of persisting proteinuria after induction treatment is not fully understood yet, but there is clear evidence that it is associated with poor kidney survival [37]. Even though the differentiation between early damage and active vasculitis remains clinical challenging. The high prevalence of persistent proteinuria more likely reflects incomplete treatment response due to early structural damage rather than ongoing active vasculitis. It is well described that specific histological patterns such as high-degree sclerosis and/or IFTA reflect a chronic kidney damage and are associated with poor renal outcomes [7, 38, 39]. On the other side, especially a high number of normal glomeruli may confer a reno-protective function [7, 38]. Previously it was already reported that chronic and acute tubulointerstitial lesions predict eGFR course after twelve months [40]. Additionally, kidney biopsy findings indicating a high level of chronic lesions as well as vascular sclerosis scores have already been used to estimate treatment resistance [16].

Also elevated serum creatinine levels seem to be associated with treatment resistance [41].

Therefore, the number of glomeruli with sclerosis could be used as an additional tool to identify patients at a high risk for incomplete renal recovery.

The current EULAR recommendations on AAV management propose an immunosuppressive maintenance therapy over a period of at least 24–48 months [14]. For patients with renal-limited disease manifestations exhibiting chronic damage such as high-degree IFTA on biopsy, our data suggest that earlier treatment withdrawal might be considered to reduce the risk of treatment-associated side effects. Importantly, even if we conducted a thoroughly analysis procedure using different multivariate models, model selection and correction for several important covariates, we were not able to include detailed ANCA autoantibody levels in our multivariate models. Considering the predictive value of increased ANCA levels for relapse events, this represents an important limitation that may affect the external validity of our results. Therefore, besides a prospective validation, further studies need to clarify the relevance of ANCA levels when relying on histological features for relapse prediction. Additional limitations of our study mainly relate to its retrospective character and a possible center bias. However, key strengths of our study include its multicenter design, large patient cohort, extended follow-up period, and different relapse definition compared to prior studies.

In conclusion, our findings suggest that moderate-to high-degree IFTA is a reliable predictor for overall and severe relapse events. These results highlight the potential of histopathological parameters at baseline to guide individualized treatment strategies, including the possibility of shortening or reducing the intensity of maintenance immunosuppression in selected patients. However, prospective validation taking into account the course of antibody concentration is essential before these insights can be translated into clinical practice.

## Supplementary Information

Below is the link to the electronic supplementary material.

Supplementary file1 (DOCX 61 KB)

## Data Availability

All data supporting the findings of this study are available within the paper and its Supplement. Individual patient data are not openly available due to sensitivity reasons but may be available from the corresponding author upon reasonable request.
