# Supplementary material for: Prediction of relapses in patients with small vessel vasculitides: a multicenter cohort study on histopathological risk patterns
Source: Rheumatol Int. 2025 Dec 2;46(1):5. doi: 10.1007/s00296-025-06049-1 (PMC12672636; doi:10.1007/s00296-025-06049-1)
Supplement: Supplementary file 1 — Supplementary file1 (DOCX 61 KB) [file 296_2025_6049_MOESM1_ESM.docx]

**Supplemental figure 1:** Study cohort, biopsy information and distribution of relapse patterns. AAV = ANCA-associated vasculitis, IFTA = interstitial fibrosis and tubular atrophy.


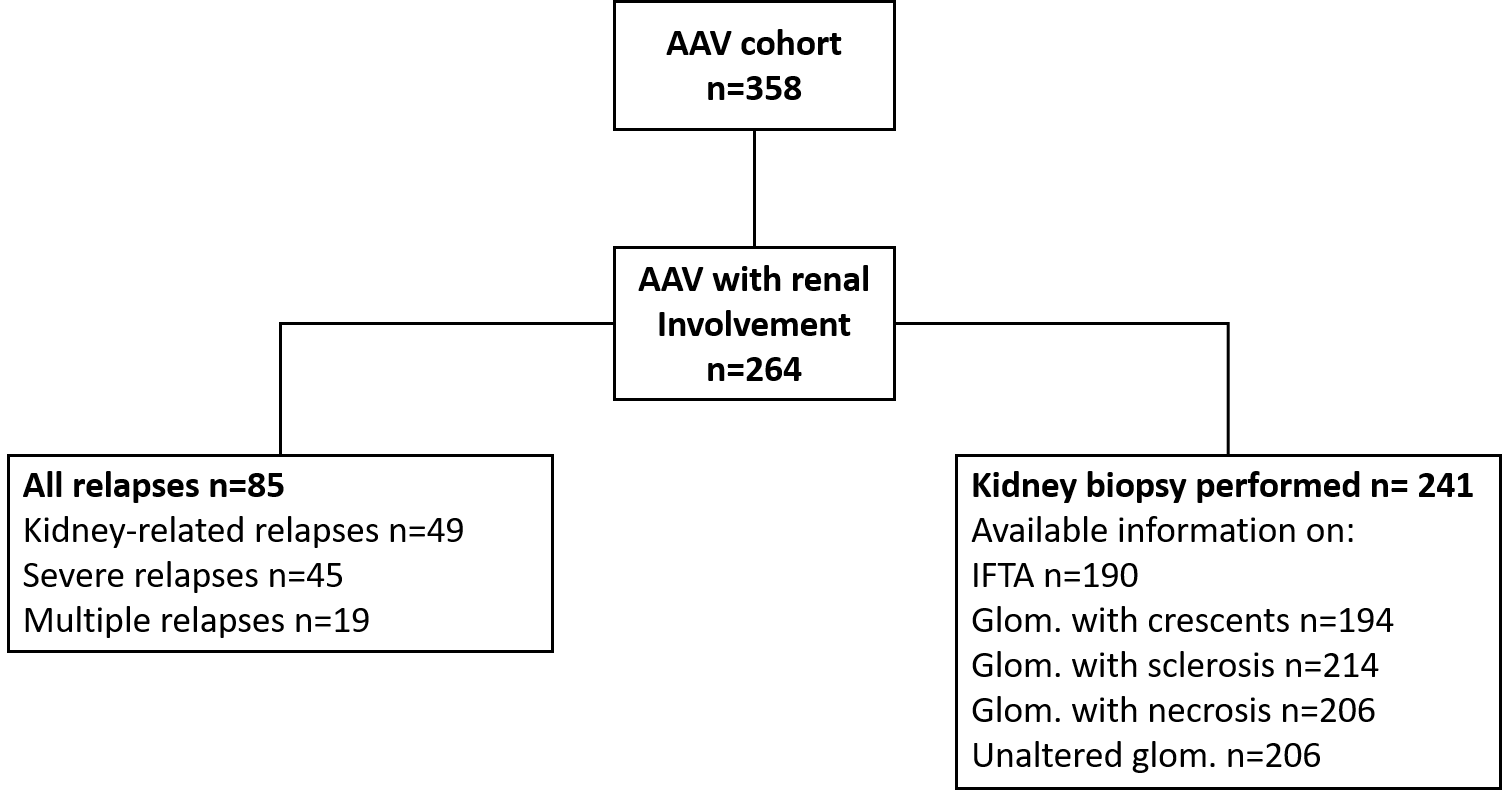


**Supplemental table 1**: Distribution for baseline predictors in relapsing and non-relapsing patients. Percentage refers to column (non-relapsing, relapsing). GPA = granulomatosis with polyangiitis, MPA = microscopic polyangiitis, CKD = chronic kidney disease, ENT = ear, nose and throat, IFTA = interstitial fibrosis and tubular atrophy, CYC = cyclophosphamide, RTX = rituximab.

| **Predictors (n=264)** | **Non-relapsing,**  **n = 179** | **Relapsing,**  **n = 85** | **P value** |
| --- | --- | --- | --- |
| GPA | 89 (49.7) | 54 (63.5) | 0.049* |
| MPA | 87 (48.6) | 31 (36.5) | 0.085 |
| Smoker | 26 (14.5) | 18 (21.2) | 0.239 |
| Male gender | 90 (50.3) | 55 (64.7) | 0.039* |
| Documented Infection | 64 (35.8) | 38 (44.7) | 0.208 |
| **BVAS categories** | | | |
| General | 98 (54.7) | 58 (68.2) | 0.051 |
| Cutaneous | 18 (10.1) | 15 (17.6) | 0.123 |
| Mucous membranes/eyes | 19 (10.6) | 16 (18.8) | 0.100 |
| ENT | 45 (25.1) | 33 (38.8) | 0.033* |
| Chest | 87 (48.6) | 42 (49.4) | 1.000 |
| Cardiovascular | 6 (3.4) | 5 (5.9) | 0.528 |
| Abdominal | 3 (16.8) | 6 (7.1) | 0.033* |
| Nervous system | 26 (14.5) | 12 (14.1) | 1.000 |
| **Pre-existing comorbidities** | | | |
| Diabetes | 19 (10.6) | 10 (11.8) | 0.945 |
| Hypertension | 102 (57.0) | 46 (54.1) | 0.760 |
| Coronary disease | 26 (14.5) | 8 (9.4) | 0.336 |
| CKD | 29 (16.2) | 5 (5.9) | 0.032* |
| **Induction treatment** | | | |
| CYC | 128 (71.5) | 69 (81.2) | 0.125 |
| RTX | 49 (27.4) | 10 (11.8) | 0.007** |
| Plasmapheresis | 42 (23.5) | 22 (25.9) | 0.783 |
| **Kidney biopsy information (n=241)** | **Non-relapsing,**  **n = 160, (%)** | **Relapsing,**  **n = 81, (%)** |  |
| IFTA >25% | 49 (30.6) | 14 (17.3) | 0.046* |
| IFTA ≥50% | 28 (17.5) | 7 (8.6) | 0.118 |
| Glom. sclerosis >25% | 62 (38.75) | 24 (29.6) | 0.155 |
| Glom. sclerosis ≥50% | 30 (18.8) | 11 (13.6) | 0.362 |
| Glom. necrosis >25% | 40 (25.0) | 24 (29.6) | 0.721 |
| Glom. necrosis ≥50% | 13 (8.1) | 10 (12.3) | 0.498 |
| Glom. crescents >25% | 28 (17.5) | 21 (25.9) | 0.250 |
| Glom. crescents ≥50% | 12 (7.5) | 12 (14.8) | 0.158 |
| Glom. unaltered ≥50% | 57 (35.6) | 33 (40.7) | 0.663 |

**Supplemental table 2**: Univariate Cox regression analysis for overall relapse events. GPA = granulomatosis with polyangiitis, MPA = microscopic polyangiitis, CKD = chronic kidney disease, ENT = ear, nose and throat, IFTA = interstitial fibrosis and tubular atrophy, CYC = cyclophosphamide, RTX = rituximab, HR = hazard ratio.

| **Predictors** | **HR** | **95%-CI** | **P value** |
| --- | --- | --- | --- |
| GPA | 1.474 | 0.947 - 2.294 | 0.086 |
| MPA | 0.686 | 0.441 - 1.068 | 0.095 |
| Smoker | 1.528 | 0.907 - 2.575 | 0.111 |
| Male | 1.515 | 0.97 - 2.365 | 0.068 |
| Documented infection | 1.122 | 0.729 - 1.726 | 0.601 |
| **Kidney biopsy information** | | | |
| IFTA >25% | 0.483 | 0.266 - 0.877 | 0.017* |
| IFTA ≥50% | 0.427 | 0.194 - 0.94 | 0.035* |
| Glom. sclerosis >25% | 0.803 | 0.491 - 1.313 | 0.382 |
| Glom. sclerosis ≥50% | 0.734 | 0.386 - 1.395 | 0.345 |
| Glom. necrosis >25% | 0.873 | 0.532 - 1.43 | 0.589 |
| Glom. necrosis ≥50% | 0.888 | 0.45 - 1.751 | 0.732 |
| Glom. crescents >25% | 1.276 | 0.762 - 2.135 | 0.354 |
| Glom. crescents ≥50% | 1.323 | 0.707 - 2.476 | 0.382 |
| Glom. unaltered ≥50% | 1.343 | 0.838 - 2.153 | 0.221 |
| **BVAS categories** | | | |
| General | 1.544 | 0.978 - 2.439 | 0.062 |
| Cutaneous | 1.539 | 0.88 - 2.692 | 0.131 |
| Mucous/Membranes/eyes | 1.823 | 1.057 - 3.146 | 0.031* |
| ENT | 1.466 | 0.946 - 2.272 | 0.087 |
| Chest | 1.025 | 0.669 - 1.568 | 0.911 |
| Cardiovascular | 1.782 | 0.721 - 4.404 | 0.211 |
| Abdominal | 1.236 | 0.536 - 2.852 | 0.620 |
| Nervous system | 1.179 | 0.639 - 2.175 | 0.599 |
| **Pre-existing comorbidities** | | | |
| Diabetes | 1.123 | 0.577 - 2.182 | 0.733 |
| Hypertension | 0.805 | 0.523 - 1.238 | 0.323 |
| Coronary disease | 0.941 | 0.453 - 1.955 | 0.872 |
| CKD | 0.430 | 0.174 - 1.062 | 0.067 |
| **Induction treatment** | | | |
| CYC | 1.034 | 0.596 - 1.794 | 0.905 |
| RTX | 0.688 | 0.353 - 1.341 | 0.272 |
| Plasmapheresis | 0.912 | 0.56 - 1.485 | 0.711 |
